# Comparison of 2-year clinical and radiological outcomes between volar plating and external fixation with additional K-wires for AO Type C distal radius fractures

**DOI:** 10.3906/sag-1811-73

**Published:** 2019-10-24

**Authors:** Özgür ÇİÇEKLİ, Erhan ŞÜKÜR, Alauddin KOCHAİ, Levent BAYAM, İzzet BİNGÖL, Uğur ÖZDEMİR

**Affiliations:** 1 Department of Orthopaedic Surgery, Sakarya University Training and Research Hospital, Sakarya Turkey; 2 Department of Orthopaedic Surgery, 29 Mayıs State Hospital, Ankara Turkey

**Keywords:** Distal radius fracture, intraarticular, volar plate, external fixator

## Abstract

**Background/aim:**

Surgical treatment of distal intraarticular radius fractures remains controversial. Our aim was to compare the clinical and radiological outcomes between volar plating (VP) and external fixation (EF) for distal intraarticular radius fractures two years postoperatively.

**Materials and methods:**

This retrospective study included 59 patients with 62 intraarticular AO Type C distal radius fractures. We distinguished two groups: patients treated with internal fixation (volar locking plate, VP group: 41 fractures), and patients treated with an external fixator and K-wires (EF group: 21 fractures). The clinical assessment included range of motion, grip strength, disability of the arm, shoulder, and hand (DASH), and visual analog scale scores. Radiological measurements comprised flexion and extension, radial volar tilt, inclination, height, shortening, and ulnar variance.

**Results:**

Postoperative grip strength and flexion angles were better after VP (P = 0.004, P = 0.009), but there was no difference in DASH scores (P = 0.341). Radial inclination was significantly different compared to that of the uninjured hand after VP (P = 0.0183), but not EF (P = 0.11).

**Conclusion:**

VP and EF result in similar clinical and radiological outcomes after 2 years. Function is not restored to the functionality of the contralateral and noninjured hand.

## 1. Introduction

The distal radius is the most common fracture site in the upper extremity. Distal radius fractures represent 75% of forearm fractures [1,2] and 17% of all fractures [1,3,4]. Distal radius fractures may occur as a result of either a high-energy trauma in a younger population or a lowenergy trauma, such as a fall on the outstretched hand, in the elderly. In the latter group, increasing life expectancy, population aging, and the subsequently higher prevalence of osteoporosis have resulted in rising overall incidences of distal radius fractures, in reports to a degree of 17% to 100% over the past three to four decades [1,5,6]. While extraarticular fractures are mostly treated nonsurgically, displaced intraarticular distal radius fractures usually require surgical intervention. Anatomic reduction and stable fixation of displaced intraarticular distal radius fractures are difficult to obtain, and poor outcomes are common [7–10]. Various surgical procedures have been described, but stabilization with a volar locking plate or an external fixator with additional K-wires are commonly used techniques [7,10–14]. Although these two methods have been previously compared in the literature, their distinct advantages and disadvantages have not been clearly established so far [7,11–13].

The aim of the current study was to compare the radiological and clinical outcomes after volar plating (VP) and external fixation with an external fixator and K-wires (EF) in distal intraarticular radius fractures, and to examine any potential relationship between radiological parameters and clinical outcome. We hypothesized that EF would lead to similar radiological and clinical outcomes as VP after an at least 24-month follow-up.

## 2. Materials and methods

Informed written consent was obtained from all participants, and the study protocol was approved by the Ethics Committee of Sakarya University (reference number 71522473/050.01.04/90).

In this retrospective cohort study, we reviewed the medical records of patients with distal radius fractures treated at our university hospital between October 2015 and June 2016. We identified 516 patients with distal radius fractures. The inclusion criteria for this study were complete intraarticular (Arbeitsgemeinschaft für Osteosynthesefragen [AO] type C) fractures [15], fixation with either volar locking plate or external fixator and K-wires, and a follow-up period of at least 24 months. The exclusion criteria were additional fractures of the injured arm, skeletally immature patients, and Gustilo–Anderson type II and III open fractures [16]. Type II and III open fractures usually required additional procedures before permanent fixation, in contrast with type I fractures. The groups were more homogenized through the exclusion of type II and III open fractures. Of the patients with radius fractures we had initially identified, 405 had been treated conservatively. The remaining 111 patients had been treated surgically. After applying the remaining inclusion and above exclusion criteria, 62 distal radius fractures in 59 patients were included in this study. Patients were divided into two groups: one group had undergone open reduction and internal fixation using a volar plate (VP group), and the other group had undergone EF with an external fixator and K-wires (EF group).

### 2.1. Surgical techniques

**Figure 1:**
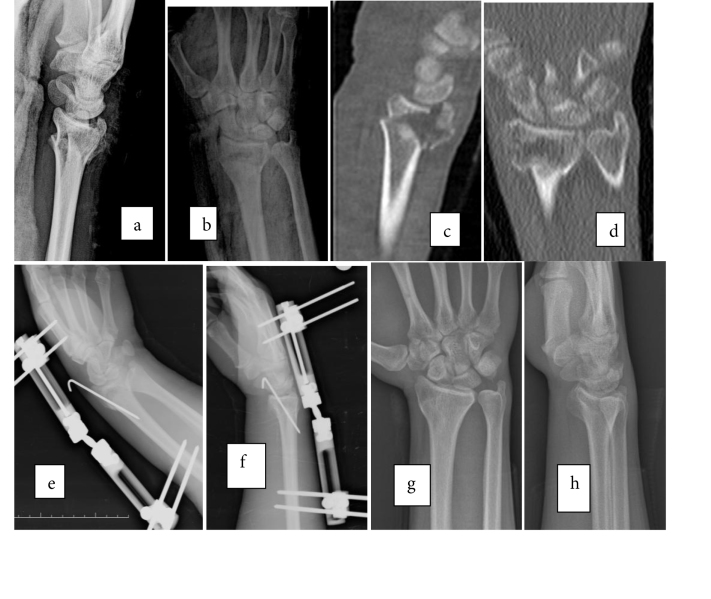
A 23-year-old male patient with a right intraarticular distal radius fracture, treated with an external fixator and a K-wire: a) and b) preoperative AP and lateral radiographies; c) and d) preoperative computed tomography (CT) scans; e) and f) immediately postoperative; and g) and h) 2-year follow-up AP and lateral radiographies.

The external fixator (Figure 1) was applied with 2 dorsolateral incisions over the radius to avoid neurovascular damage. Two threaded 3.0-mm pins were inserted in a dorsolateral direction. In the next step, 2 small dorsolateral incisions were made on the second metacarpal bone and 2 threaded 2.5-mm pins inserted. After fracture reduction was achieved under fluoroscopy control, the articular fragment was fixated with 1.6 mm K-wires. The external fixator frame (Dynamic Angled Clamp Wrist Fixator; TST Medical Devices, İstanbul, Turkey) and spanning bars were then mounted and stabilized with the clamps (Figure 1). Dorsal arthrotomy and open reduction were considered to be indicated in cases of inadequate reduction, defined as a >2.0 mm articular step-off, >5.0 mm radial shortening, or >10° dorsal angulation [1,10,17,18]. Dorsal arthrotomy was applied to only 4 patients out of 21 EF. Articular stepoff of these patients was measured by using computed tomography (CT) before operating and by guidance of fluoroscopy intraoperatively.

**Figure 2:**
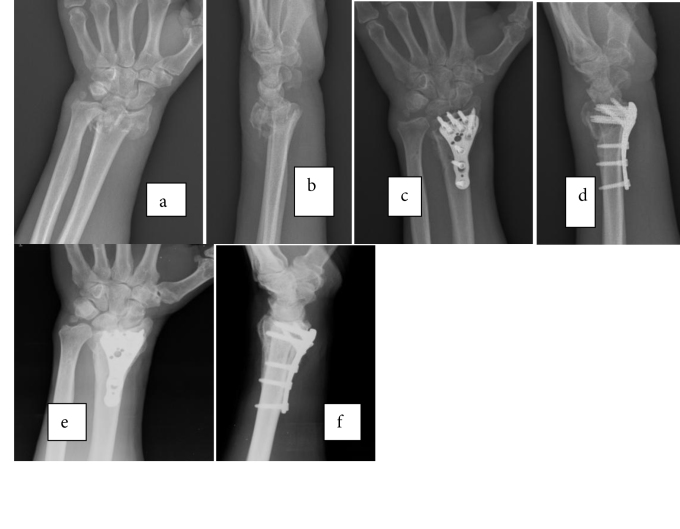
A 54-year-old man with a left displaced intraarticular distal radius fracture, treated with volar plating: a) and b) preoperative; c) and d) immediately postoperative; e) and f) 2-year follow-up AP and lateral radiographies.

VP (Figure 2) was performed via a modified Henry approach. The flexor carpi radialis tendon sheath was incised longitudinally and the tendon retracted to the ulnar side. The flexor pollicis longus muscle was retracted radially and the pronator quadratus muscle incised to expose the radius fracture. Fracture fragments were reduced and temporarily fixated with a 1.6-mm K-wire under fluoroscopy control to ensure proper alignment. Next, the volar plate (Distal Radius Volar Plate; TST Medical Devices, İstanbul, Turkey) was applied and fixated with locking screws (Figure 2). The pronator quadratus muscle was repaired and the stabilizing wires were removed prior to skin closure.

### 2.3. Clinical assessment

All patients had a minimum 2-year follow-up and regular clinical assessments during this period. Patients were evaluated by the same observer for their active range of motion (ROM), disabilities of the arm, shoulder, and hand (DASH) score, and grip strength. Wrist ROM was measured using a standard goniometer. Wrist flexion and extension and radial and ulnar deviation were measured for both the injured and uninjured sides. DASH has been validated for the Turkish language; scoring ranges from 0 (no disability) to 100 (maximum disability). Grip strength was measured in kilograms using a hydraulic hand dynamometer (Saehan Hydraulic Hand Dynamometer; Saehan Corporation, Changwon, South Korea) on both the injured and uninjured sides. The patients’ satisfaction with the treatment results and presence of pain were established and documented. The visual analogue scale (VAS) as a validated, subjective measure was used to assess pain after surgery (scores range from 0 = no pain to 100 = worst pain possible) [19]. We also assessed and recorded complex regional pain syndrome (CRPS).

### 2.4. Radiological outcomes

All measurements were based on standard lateral and anteroposterior views after at least 2 years of follow-up. Volar tilt, radial inclination, ulnar variance, radial height, and articular step-off were analyzed on the radiographs using a specific program within the Picture Archiving and Communication System (KarMed PACS, Kardelen Medical Software, Mersin, Turkey). A radial inclination >15°, radial shortening <5 mm, sagittal tilt between 15° dorsal and 20° volar, and an intraarticular step-off <2 mm were our criteria for an acceptable reduction (normal volar tilt is considered to be 11° ± 5°; normal radial inclination, ulnar variance and radial height are 22° ± 3°, 0.7 ± 1.5 mm, and 14 ± 1 mm, respectively [1,20]. All measurements were recorded for both the injured and the uninjured wrists in each patient.

### 2.5. Other outcomes

Complications such as median nerve injury, infection, and vascular injuries were also recorded, as well as the need for additional surgical procedures.

### 2.6. Statistical analysis

An online statistical program, Social Science Statistics (Stangroom J [2018]) was used for statistical calculations and descriptive analysis. To examine the association and correlation between two parametric data (continuous variables such as DASH scores, VAS scores, and radiological measurements), Pearson’s correlation was used, but when one or two of the variables were on an ordinal scale, such as fracture type or complication rate, Spearman’s correlation was used. The independent t-test was used to compare parametric data between the two groups. A P-value of ≤0.05 was considered statistically significant.

## 3. Results

We analyzed the clinical and radiological outcomes of 62 distal radius fractures in 59 patients (38 men and 21 women). There were 41 distal radius fractures in the VP group and 21 in the EF group. The two groups were not significantly different with regard to their demographic and other characteristics (Table 1). In the VP group, 9 fractures were AO type C1 fractures, 24 were AO type C2 fractures, and 8 were AO type C3 fractures, whereas in the EF group 6 fractures were AO type C1 fractures, 10 were AO type C2 fractures, and 5 were AO type C3 fractures (Table 2). There were 11 patients of type I open fractures, 5 in the EF group and 6 in the VP group. One patient had bilateral distal radius fractures and was treated with VP for both. Another patient with bilateral fractures was treated with EF for both. One patient had revision surgery where EF was changed to VP. This case was excluded from the statistical analysis. Mean time of removing the external fixator was 6.77 weeks (range: 5–8 weeks).

**Table 1:** Demographic data of 59 patients treated with either volar plating (VP) or external fixation (EF) for 62 intraarticular distal radius fractures and mean follow-up period.

	Number of radius fractures (n)	Number of patients (n)	Malepatients (n)	Femalepatients (n)	Mean Age(y) (range)	Mean follow-up period (months)
VP group	41	40	25	15	42.6 (22–78)	28.02
EF group	21	19	13	6	47.2 (21–73)	27.43
All patients	62	59	38	21	46.3 (21–78)	27.85

**Table 2:** AO classification of distal radius fractures for volar plating (VP) and external fixation (EF) groups.

	C1 (n)	C2 (n)	C3 (n)	Total (n)
VP group	9	24	8	41
EF group	6	10	5	21

Postoperative grip strength, flexion angles, and ulnar deviation for the VP group were better, but there was no difference in postoperative DASH scores between the two groups (Table 3). Furthermore, there were no statistically significant differences in postoperative radial height, radial inclination, ulnar variance, and volar tilt between the two groups (Table 3). The mean radial height for the VP group was 11.1 mm; for the EF group, 11.0 mm.

**Table 3:** T-test results for relationship between the volar plating (VP) and external fixation (EF) group (total of 62 distal radial fractures) outcomes with regard to radiological measurements, disabilities of the arm, hand, and shoulder (DASH) score, and grip strength (p1) and comparison with the uninjured hand/wrist for all patients (p).

	Volar plate			External fixation			p1
	Operated (n = 41)	Uninjured(n = 41)	p	Operated(n = 21)	Uninjured(n = 21)	P	
Flexion (°)	60.7 ± 14.5	73.4 ± 11.2	0.0004	51.5 ± 13.6	68.7 ± 10.1	0.0001	0.009
Extension (°)	53.6 ± 14.1	63.6 ± 6.1	0.0007	46.8 ± 17.8	63.4 ± 7.2	0.002	0.052
Ulnar deviation (°)	19.5 ± 6.6	24.7 ± 6.5	0.0003	16.3 ± 6.9	21.2 ± 5.1	0.01	0.043
Radial deviation (°)	15.4 ± 5.3	21.3 ± 6.2	0.002	12.7 ± 5.4	21.5 ± 5.7	0.003	0.385
DASH scores	16.3 ± 11.3	5.3 ± 1.5	0.0001	17.6 ± 11.9	6.2 ± 2.1	0.0002	0.341
Grip strength (kg)	49.7 ± 20.4	70.4 ± 19.7	0.0001	35.1 ± 18.7	58 ± 12.6	0.0008	0.004
Radial inclination (°)	19.1 ± 4.3	20.7 ± 1.9	0.02	19.3 ± 4.5	21.1 ± 3.2	0.11	0.435
Volar tilt (°)	5.1 ± 2.9	9.9 ± 1.6	0.0003	3.1 ± 2.1	8.4 ± 1.8	0.0001	0.11
Radial height (mm)	11.1 ± 2.2	12.2 ± 1.1	0.003	11.0 ± 1.8	12.1 ± 1	0.002	0.407
Ulnar variance (mm)	0.16 ± 0.8	0.1 ± 0.2	0.32	0.22 ± 1.5	0.12 ± 0.1	0.45	0.1

In patients of both groups, the comparison of the fractured hand/wrist postoperatively with the uninjured hand/wrist showed that there were statistically significant differences for grip strength, flexion and extension angles, radial and ulnar deviations, radial height, and volar tilt (Table 3). Radial inclination showed a significant difference between the fractured and the uninjured sides in patients of the VP group, but not in patients of the EF group. The mean volar tilt was 5.1° in the wrist which had been operated on vs 9.9° in the uninjured wrist in the VP group, and 3.1° vs 8.4°, respectively, in the EF group (Table 3).

Table 3 shows a strong correlation between the DASH and VAS scores (r = 0.929, r2 = 0.864). There was a weak negative correlation between the DASH score and radial tilt, inclination, and shortening, and a weak positive correlation between grip strength and radial tilt, as well as shortening (Table 4).

**Table 4:** Pearson test results for relationships between parametric data in 62 distal radial fractures: disabilities of the arm, hand, and shoulder (DASH) score, visual analog scale (VAS) score, grip strength, radial tilt, inclination, and shortening regardless of the fixation method.

	r-value	r2	Correlation
DASH score and VAS score	0.929	0.864	strong positive
Radial volar tilt and DASH score	–0.096	0.0092	weak negative
Radial volar tilt and grip strength	0.13	0.016	weak positive
Radial inclination and DASH score	–0.087	0.0076	weak negative
Radial shortening and DASH score	–0.034	0.0012	weak negative
Radial shortening and grip strength	0.154	0.024	weak positive

Complication rate of patients during the postoperative period was 24% (n = 15). With regard to complications, there were 3 superficial infections and 2 median nerve entrapments in the VP group, and 1 superficial infection and no median nerve entrapments in the EF group. No vascular injuries occurred in either group. There were statistically significantly more CRPSs in the EF (n = 8) group compared with the VP (n = 6) group (Table 5). No correlations were observed between fracture type and DASH score, fracture type, and CRPS (Table 5).

**Table 5:** Spearman test results for the relationship between nonparametric data in 62 distal radial fractures: volar plating (VP)/external fixation (EF) group, fracture type, disabilities of the arm, hand, and shoulder (DASH) score, open and closed fractures compared with complex regional pain syndrome (CPRS).

				r	P
CRPS	VP (n = 41)	6		0.249	0.049	EF (n = 21)	8		Open (n = 13)	4		0.199	0.118	Closed (n = 49)	10	
	Fracture type	C1 (n = 15)	C2 (n = 34)			C3 (n = 13)							
	DASH score	16.1	16.7	18.2	0.043	0.074
	CRPS	3	6	5	0.138	0.28

## 4. Discussion

The most important finding of this study was that the clinical outcomes were similar for the VP and EF group 2 years postoperatively. Although grip strength, wrist flexion, and ulnar deviation angles were better in the VP group, the DASH scores were not different between the two groups. Another important finding was that radial inclination was statistically significantly different between the operated and the uninjured sides in the VP group, but not in the EF group. This means that the external fixator maintained this angle better than the volar plate. There were no significant differences between radial volar tilt angle and clinical outcomes in both groups.

### 4.1. Clinical outcomes

Navarro et al. randomized 140 patients with distal radius fractures into a VP and EF group and reported no difference in the DASH scores [21]. However, they included intraand extraarticular fractures in patients aged between 50 and 74 years. Shukla et al. in their prospective randomized study of displaced intraarticular fractures reported better outcomes in the EF group after 1 year of follow-up [17]. They found excellent results for patients under 50 years treated with EF. Roh et al. observed better short-term clinical outcomes in the VP group than in the EF group and similar outcomes after 1 year [7]. They concluded that VP was better than EF, especially for patients under 54 years with AO type C2 and C3 distal radius fractures. Kumbaraci et al. reviewed the results of 69 patients with intraarticular distal radius fractures and claimed that VP had better clinical outcomes compared to EF [7]. Some prospective randomized studies showed better outcomes postoperatively for VP, especially in the first 3 months, although the outcomes for VP and EF were similar after the first year [12,13]. These authors also recommended VP for younger patients. In a recent study, Drobetz et al. suggested better clinical and functional outcomes with VP compared to other treatment modalities, but they analyzed data from both intra- and extraarticular fractures [22]. In our study, no difference was found in the DASH scores between the two techniques, and the clinical outcomes with either technique were not able to match the functional capacity of the uninjured wrist and hand.

Grip strength was better for the VP group compared to the EF group in the short and long term, while wrist flexion–extension angles were similar after 1 year according to the prospective randomized study of Jeudy at al. [12]. Grewal at al. reported better results for grip strength and wrist ROM for VP compared to EF in the early postoperative period, but had similar clinical results after 1 year [13]. Kumbaraci et al. established similar results for grip strength but significantly better pronation and flexion in VP [11]. Although better grip strength was documented during the early postoperative period in the VP group, there was no difference after 1 year between the VP and EF group in the studies by Navarro et al. and Roh et al. [7,21]. Neither did they find a difference in wrist ROM. At the same time, Shukla et al. reported better grip strength and wrist ROM for the EF group after 1 year [17]. In our study, the better grip strength and wrist ROM in the VP group did not affect the overall function of the wrist.

### 4.2. Radiological outcomes

In a recent metaanalysis, Chaudhry et al. reported no differences between VP and K-wires when assessing the radiological outcomes only [14]. Grewal et al. showed similar radiological outcomes between VP and EF in the early postoperative period except for a slightly better result for volar tilt in the VP group [13]. Although better radiological outcomes were reported for VP in several studies [11,21–24], Shukla et al. [17] did not find any differences. Roh et al. [7] also reported no significant differences with respect to volar tilt or radial inclination, but found more favorable results for ulnar variance in the VP group. Dario et al. [1] suggested that ulnar variance and volar tilt are the most important radiological parameters that have to be restored in order to obtain a good functional outcome in distal radius fractures. Mignemi et al. claimed that, in addition, radial inclination, radial height, and articular congruence are important factors in determining the long-term outcome [10]. Şenel et al. [25] reported satisfactory clinical and radiological outcomes for AO type C distal radius fractures.

The better radiological measurements in the VP group in our study were not statistically significant. Radial volar tilt could not be restored to the degree found on the uninjured side with either technique in our patients, but radial inclination was better restored in the EF group and did not differ significantly from the uninjured wrist. We found a weak negative correlation between volar tilt and DASH score and a weak positive correlation between volar tilt and grip strength.

### 4.3. Complication rates

The complications in VP and EF are known to differ in both quality and quantity [24]. Higher rates of neuritis, implant failure, and infection have been recorded for EF, while tendon complications and early implant removal have been observed more frequently with VP in the studies by Jorge-Mora et al. and Margaliot [24,26]. Leung et al. found a lower rate of secondary osteoarthritis [27] and Kumbaraci et al. [11] a lower complication rate overall in the VP group. However, similar total complication rates were reported by Navarro et al. [21] and Shukla et al. [17] for both methods. Only superficial wound infections occurred more frequently in the K-wire only group, whereas other complication rates were similar between the two groups according to the metaanalysis by Chaudhry et al. [14]. Jeudy et al. reported higher complication rates in the first 6 months for the EF group, while there was no difference after 1 year between EF and VP [12]. Roh et al. found a higher complication rate for EF, but no significant difference for CRPS compared to VP [7]. Some studies suggested that CRPS is more likely to occur after EF [7,28], while others showed no difference [29]. Similarly, we observed more CRPS in the EF group.

### 4.4. Strengths and limitations of this study

The comparison of both functional and radiological outcome parameters for the two surgical methods in the treatment of intraarticular fractures over a period of 2 years was the main strength of our study. The postoperative parameters were also compared with the uninjured side in each patient for both methods. We consider the large range in patients’ ages to be a limitation of our study. A more homogenous group with regard to age could improve the strength of future studies. The efficacy of osteoporosis on fixation techniques and outcomes of distal radius fractures was not studied. This was another limitation. Furthermore, investigating the effect of socioeconomic factors on clinical outcomes might help to explain the findings on self-reported wrist pain.

In conclusion, both VP and EF were effective fixation methods for intraarticular distal radius fractures, resulting in similar functional and radiological outcomes. Despite satisfactory clinical and radiological outcomes, the overall functional outcome with either method was not as good as the function of the uninjured wrist in our patients.
